# Contaminated Soil and Transmission of Influenza Virus (H5N1)

**DOI:** 10.3201/eid1809.120402

**Published:** 2012-09

**Authors:** Ramona A. Gutiérrez, Philippe Buchy

**Affiliations:** Institut Pasteur in Cambodia, Phnom Penh, Cambodia

**Keywords:** influenza, H5N1 virus, Southeast Asia, environment, soil, poultry, viruses, contamination, Cambodia

**To the Editor:** Highly pathogenic avian influenza (HPAI) virus (H5N1) has been responsible for 603 confirmed human cases worldwide, including 356 that resulted in death, and for >7,000 epizootic outbreaks (*1*,*2*). Direct contact between hosts is the main mechanism of transmission for avian influenza viruses, but the possible role of the environment as a source of HPAI virus (H5N1) infection has been rarely studied, particularly in the context of countries where the virus is enzootic or epizootic (*3*–*7*). To determine if contaminated soil contributes to the transmission cycle of HPAI virus (H5N1), we used experimental and simulated field conditions to assess possible transmission in chickens.

All experiments were conducted by using HPAI virus (H5N1) strain A/chicken/Cambodia/LC1AL/2007 (GenBank accession nos. HQ200574–HQ200581). All animal experiments were conducted in the biosafety level 3 laboratory of Institut Pasteur in Cambodia (IPC), in compliance with the European Community 86/609/CEE directive and approved by the Animal Ethics Committee of IPC (permit: AEC/IPC/003/2010). Specific pathogen–free (SPF) chickens were provided by the National Veterinary Research Institute of Cambodia.

We used 3 types of soil: 1) sandy topsoil collected from around rice fields in Phnom Penh Province, Cambodia; 2) building sand purchased from a local building company; and 3) soil-based compost purchased from a local tree nursery. Physicochemical and microbiologic parameters were measured for water extracts obtained for each type of soil (Technical Appendix Table), and low- and high-dose contamination protocols (Technical Appendix Figure) were used to experimentally contaminate each soil type. In brief, we seeded the soil samples with 1–56 infectious units of contaminated feces; 1 infectious unit was defined as 1 g feces from an SPF duck mixed with 1 × 10^7.8^ 50% egg infective dose of HPAI virus (H5N1) particles. The contaminated soil was then sprinkled on the bottom of an isolator (surface area 0.2 m^2^) in which the chickens were housed. Oropharyngeal and cloacal swab samples and feathers were collected daily from the chickens and underwent quantitative reverse transcription PCR (qRT-PCR) testing targeting the H5 hemagglutinin gene (*8*). Surviving birds were killed humanely at the end of the experiments, and postmortem examination and collection of serum and organ samples were conducted on all animals. Organ samples were tested by using qRT-PCR, and serum samples were tested by using hemagglutination inhibition assay (*9*).

No clinical symptoms, deaths, or seroconversion for HPAI virus (H5N1) were observed in chickens exposed to contaminated sandy topsoil, regardless of the dose protocol used (Table). However, for building sand and soil-based compost, the high-dose contamination protocol, starting with 8 infectious units, resulted in a 100% mortality rate by day 4. Low-dose protocols, starting at 1 infectious unit, resulted in survival of all birds at day 24, with no clinical symptoms and no virus detected in the samples collected postmortem. However, seroconversion for HPAI virus (H5N1) was observed in 33% and 50% of the chickens exposed to building sand and compost, respectively (Table).

**Table:** Clinical, virologic, and serologic results obtained from chickens exposed to soils experimentally contaminated with influenza virus (H5N1)*

Protocol	Sandy topsoil (rice fields)	Building sand	Soil-based compost
Low-dose contamination			
Clinical signs	None	None	None
Mortality rate	None	None	None
Virus in postmortem samples	NA	NA	NA
Seroconversion rate	None	33% at day 24	50% at day 24
High-dose contamination			
Clinical signs	None	None	None
Mortality rate	None	100% by day 4	100% by day 4
Virus in postmortem samples	NA	Yes	Yes
Seroconversion rate	None	NA	NA

*Low-dose contamination protocol, exposure of the chickens to soil inoculated with 1 infectious unit on day 0, 2 on day 6, 4 on day 12, and 8 on day 18. High-dose contamination protocol, exposure of the chickens to soil inoculated with 8 infectious units on day 0, 12 on day 6, 16 on day 12, and 20 on day 18. In each experiment, 10–20 chickens were exposed to contaminated soils. Each experiment was repeated twice. For each experiment, a control group was established (same number of chickens exposed to noncontaminated soils; no deaths were observed in control groups). NA, not applicable.

Soil-based compost and building sand, although existing in natural settings, are not the most common substrates found in places where free-ranging poultry are raised in Cambodia. Therefore, despite the high mortality rate observed in our study after exposure to highly contaminated soils, the role of these soil types in transmission of HPAI virus (H5N1) infection to poultry or other species, including humans, appears limited when replaced in actual epizootic or enzootic field conditions. Our results also show that exposure of chickens to moderately contaminated soil may result in a protective immune response.

Sandy topsoil, on the other hand, did not allow any transmission of HPAI virus (H5N1) from the environment to chickens. This type of soil, which covers ≈40% of the rice-growing areas of Cambodia (*10*) and is abundant in neighboring countries of the Mekong region, is the most common ground on which local poultry are found wandering and the typical soil found at the sites of HPAI virus (H5N1) outbreaks. This sandy topsoil is acidic and poorly buffered, which explains the differences observed between our indirect pH measures and the direct measures reported in specialized literature (*10*). The soil’s low pH may inactivate enveloped viral particles, as well as bacteria (Technical Appendix Table).

In Cambodia, as in several other countries affected by HPAI virus (H5N1), decontamination of the environment after an outbreak is recommended by authorities; for example, disinfectants such as cresols are sprayed over environmental surfaces. However, because of resource limitations, only limited areas can be treated. Our results provide evidence that, even when abundantly contaminated, some soil types are unlikely to allow transmission of the virus to poultry and, consequently, probably not to other animals or to humans. These results suggest that limited resources could be better concentrated in high-risk areas, where the nature of the soils would be more likely to lead to poultry infection after natural contamination. These data may aid in the design of more cost-effective and solid-based decontamination measures for preventing transmission of HPAI virus (H5N1) to humans and animals.

Technical Appendix Physico-chemical and bacteriological parameters measured in soils tested, and the low- and high-dose contamination procotols used for testing.
