# Supplementary material for: Contaminated Soil and Transmission of Influenza Virus (H5N1)
Source: Emerg Infect Dis. 2012 Sep;18(9):1530–2. doi: 10.3201/eid1809.120402 (PMC3437732; doi:10.3201/eid1809.120402)
Supplement: Technical Appendix — Physico-chemical and bacteriological parameters measured in soils tested, and the low- and high-dose contamination procotols used for testing. [file 12-0402-Techapp-s1.pdf]

Article DOI: http://dx.doi.org/10.3201/eid1809.120402

# Contaminated Soil and Transmission of Influenza Virus (H5N1)

## Technical Appendix

Technical Appendix Table. Physico-chemical and bacteriological parameters measured in the soils tested

| | | Sandy topsoil (rice fields) | Building sand | Soil-based compost |
|---|---|---|---|---|
| Microbiological analysis | Total aerobic plate count at 37°C, 24h (CFU/mL) | $2.00 \times 10^2$ | $8.00 \times 10^4$ | $2.00 \times 10^5$ |
| | Total aerobic plate count at 22°C, 72h (CFU/mL) | $6.00 \times 10^2$ | $9.00 \times 10^4$ | $3.40 \times 10^5$ |
| | Total Coliforms (CFU/100mL) | 30 | $3.00 \times 10^6$ | $7.20 \times 10^6$ |
| | Thermotolerant Coliforms (CFU/100mL) | 10 | $1.00 \times 10^6$ | $4.00 \times 10^6$ |
| | *Escherichia coli* (CFU/100mL) | <1 | $5.00 \times 10^2$ | $1.70 \times 10^4$ |
| | *Enterococcus faecalis* (CFU/100mL) | <1 | $2.00 \times 10^2$ | $2.30 \times 10^3$ |
| | Sulfite reducing anaerobes (CFU/20mL) | 4 | $6.00 \times 10^2$ | $5.00 \times 10^3$ |
| Physico-chemical analysis | Turbidity (NTU) | >100 | >100 | >100 |
| | pH | 6.4 | 6.3 | 6.3 |
| | Chloride (mg/L) | 76 | 41 | 42 |
| | Ammonia (mg/L) | 0.88 | 0.35 | 1.61 |
| | Nitrite (mg/L) | 0 | 0 | 0 |
| | Nitrate (mg/L | 1.6 | 0.22 | 0.46 |
| | Hardness (mg/L) | 7 | 11 | 9 |
| | Iron (mg/L) | 0 | 0 | 0 |

All analyses were conducted on water extracts obtained from mixing the soils with distilled water.

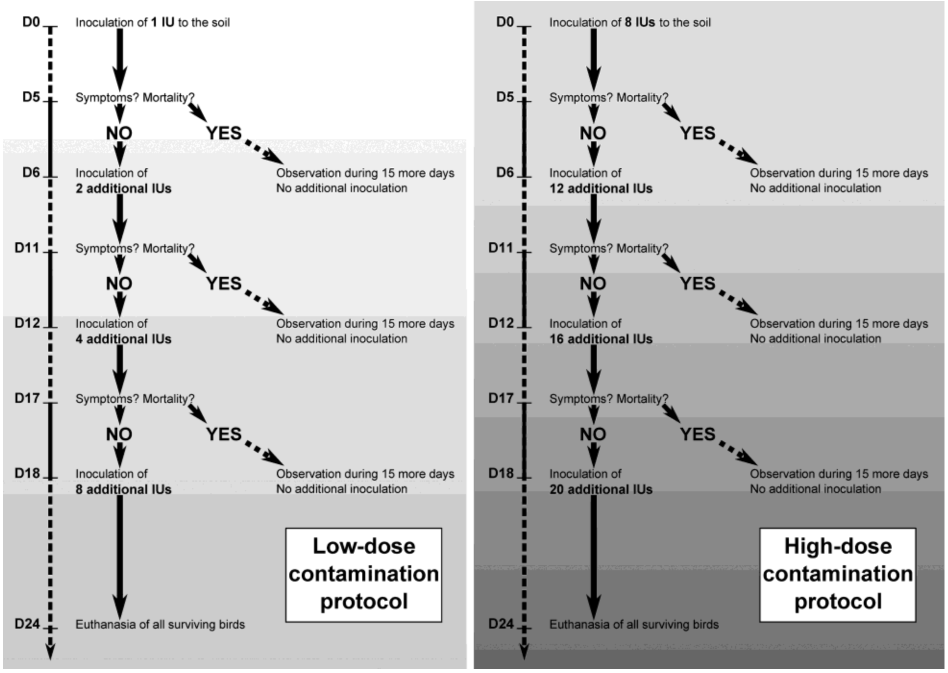


Technical Appendix Figure. Low- and high-dose contamination procotols. IU: Infectious Unit = 1g of Specific Pathogen Free ducks feces, experimentally contaminated with $10^{7.8}$ Egg Infective Dose 50% (EID50). D: Day.
